# EpiJen: a server for multistep T cell epitope prediction

**DOI:** 10.1186/1471-2105-7-131

**Published:** 2006-03-13

**Authors:** Irini A Doytchinova, Pingping Guan, Darren R Flower

**Affiliations:** 1 Edward Jenner Institute for Vaccine Research, Compton, RG20 7NN, UK

## Abstract

**Background:**

The main processing pathway for MHC class I ligands involves degradation of proteins by the proteasome, followed by transport of products by the transporter associated with antigen processing (TAP) to the endoplasmic reticulum (ER), where peptides are bound by MHC class I molecules, and then presented on the cell surface by MHCs. The whole process is modeled here using an integrated approach, which we call EpiJen. EpiJen is based on quantitative matrices, derived by the additive method, and applied successively to select epitopes. EpiJen is available free online.

**Results:**

To identify epitopes, a source protein is passed through four steps: proteasome cleavage, TAP transport, MHC binding and epitope selection. At each stage, different proportions of non-epitopes are eliminated. The final set of peptides represents no more than 5% of the whole protein sequence and will contain 85% of the true epitopes, as indicated by external validation. Compared to other integrated methods (NetCTL, WAPP and SMM), EpiJen performs best, predicting 61 of the 99 HIV epitopes used in this study.

**Conclusion:**

EpiJen is a reliable multi-step algorithm for T cell epitope prediction, which belongs to the next generation of *in silico *T cell epitope identification methods. These methods aim to reduce subsequent experimental work by improving the success rate of epitope prediction.

## Background

The accurate identification of T-cell epitopes remains a critical step in the development of subunit and peptide-based vaccines [1]. The first step of such studies is usually *in silico *prediction of potential MHC binders from the sequence of a studied protein, followed by labor-, time- and resource-consuming experiments which aim to verify the natural processing, presentation and T-cell recognition of the predicted peptides. As the veracity of initial *in silico *predictions improves, so subsequent "wet lab" work becomes faster, more efficient, and, ultimately, more successful.

The main processing pathway for Major Histocompatibility Complex (MHC) class I ligands involves degradation of proteins by the proteasome, followed by transport of the products by the transporter associated with antigen processing (TAP) to the endoplasmic reticulum (ER), where peptides are bound to MHC class I molecules, and then presented on the cell surface by MHCs. The proteasome is responsible for generating the C terminus but not the N terminus of the final presented peptide [2-5]. The proteasome is a multimeric proteinase with three active sites: a site with trypsin-like activity (cleavage after basic residues), one with chymotrypsin-like activity (cleavage after hydrophobic residues), and another with peptidylglutamyl-peptide hydrolytic activity (cleavage after acidic residues) [6-8]. In addition, in vertebrates there are three γ-interferon-inducible subunits that replace the constitutive subunits [9] and assemble the immunoproteasome. The immunoproteasomes have an altered hierarchy of proteosomal cleavage, enhancing cleavage after basic and hydrophobic residues and inhibiting cleavage after acidic residues [10,11]. This is in accord with the amino acid preferences for binding to MHC class I molecules at the C terminus [12].

TAP is an ATP-dependent peptide transport protein that belongs to the ATP-binding cassette (ABC) family of transporters. This family transports across membranes a wide range of molecules, from small sugars to large polypeptides [13]. There are two TAP proteins (TAP-1 and TAP-2) which form a transmembrane (TM) heterodimer. Both proteins encode one hydrophobic TM domain and one ATP-binding domain [14]. Extant experimental studies have shown that TAP prefers peptides of eight or more amino acids with hydrophobic or basic residues at the carboxy terminus [15,16]. TAP-mediated antigen presentation is important not only for cytosolic antigens but also for most epitopes within membrane/secretory proteins [17]. The TAP-dependent pathway is the principal processing route for peptides binding HLA-A1, HLA-A3, HLA-A11, HLA-A24, HLA-B15 and HLA-B27 [18-20]. Some peptides are able to access the ER via other, TAP-independent mechanisms. Examples of alleles exhibiting only partial dependence on TAP include HLA-A2, HLA-A23, HLA-B7 and HLA-B8 [21-24].

Proteins of the MHC are both polygenic (i.e. there are more than one MHC class I and MHC class II genes) and polymorphic (i.e. there are many alleles of each gene) [25]. Each class of MHC has several loci: HLA-A, HLA-B and HLA-C for class I and HLA-DR, HLA-DQ and HLA-DP for class II. MHC alleles may differ by as many as 30 amino acid substitutions, most of them are found within the binding site and are critical for the specificity of peptide binding and therefore for T cell recognition [26-28]. Such an uncommon degree of polymorphism implies a selective pressure to create and maintain it. Different polymorphic MHC alleles have different peptide specificities: each allele binds peptides exhibiting particular sequence patterns.

Successful T cell epitope prediction has always challenged bioinformatics. A wide range of computer-based algorithms have been developed to predict T-cell epitopes [29-31]. Initially, methods for direct T cell epitope prediction were developed based on amphipathicy [32], epitope pattern motifs [33], or on a combination there of [34]. These direct epitope prediction methods have relatively low accuracy [35]. Later, a broad spectrum of indirect predictive methods have been developed to predict MHC binders instead of T cell epitopes [reviewed in ref. [36]]. They are based on motif patterns [37,38], motif profiles [39,40], quantitative matrices (QM) [41-43], neural networks (ANN) [44-47], free energy scoring functions (Fresno) [48], MHC-peptide threading [49], 3D-QSAR studies [50-52] and support vector machines (SVM) [53,54]. In general, methods of this type have out-performed older methods. In the meantime, methods for the prediction of proteasome cleavage [55-57] and TAP binding [58-61] were developed. These methods attempt to model the early stages of the antigen processing pathway.

The next generation of T cell epitope identification methods will focus on integrated multi-step approaches, which subsume proteasome cleavage, TAP transport and MHC binding. The advantages of such integrated methods are higher accuracy and a lower rate of false positive predictions, although they may generate more false negative predictions due to the use of incomplete training sets or high thresholds for individual steps. Although some previous attempts have been made to combine predictive methods [57,60,62,63], true integrated methods have only recently emerged, examples include: SMM [64], NetCTL [65] and WAPP [66]. SMM stands for Stabilized Matrix Method and is a T cell epitope predictive tool based on QMs for binding to MHC class I molecules, peptide transport by TAP and proteasomal (or immunoproteasomal) cleavage of protein sequences [64]. NetCTL implements, in a combined manner, ANN-based proteasome cleavage prediction, a TAP binding QM and ANN-based MHC class I binding prediction [65]. WAPP applies proteasomal cleavage matrices, SVM-based TAP and MHC predictions as a series of successive filters [66]. All three methods emphasize the greatly reduced number of peptides which need to be tested in order to identify true epitopes; they show good accuracy for sets they have been tested with.

**Figure 1:**
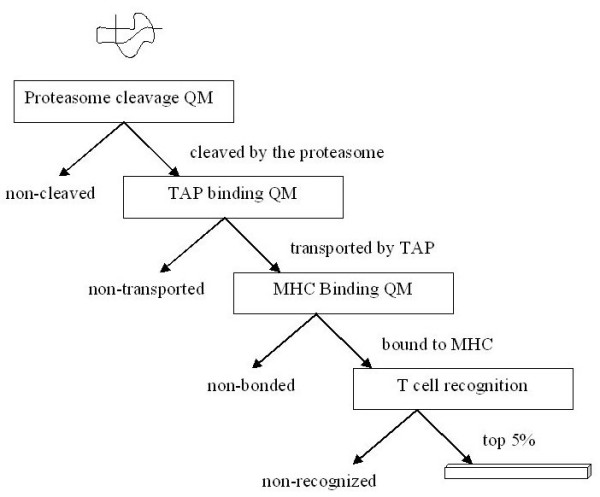
Flowchart of EpiJen.

In the present study, we develop a multi-step algorithm for T cell epitope prediction, which we call EpiJen. The method is applied to a set of overlapping peptides generated from a whole protein sequence and acts as a series of filters which successfully reduce the number of potential epitopes (Figure 1). The final set of peptides needed to be tested for epitopes rarely includes more than 5% of the whole sequence. QMs for each step were developed using the additive method [42]. Since its appearance in 2001, this method has been applied to more than 2500 peptides binding to several human and murine MHC class I and class II proteins [67] and has been shown to be both reliable and highly predictive, allowing us to design superbinders [68]. The method was also used to generate QMs for TAP binding [69] and proteasome cleavage [70]. Recently, several new models have been developed for peptide binding to human MHC alleles: HLA-A*24, HLA-B*07, HLA-B*27, HLA-B*40, HLA-B*44, HLA-B*51 and HLA-B*53 (unpublished results). Here, we combine all additive method human models and make them publicly available via the EpiJen server for T cell epitope prediction [71]. The performance of EpiJen is tested using external sets of known T cell epitopes and is compared with the predictions made by SMM, NetCTL and WAPP methods.

## Results

### EpiJen step one: proteasome cleavage

The dataflow in EpiJen is shown in Figure 1. Initially, the protein is chopped into overlapping decamers and processed by a proteasome cleavage QM. A previously derived and tested p1p1' model, as described in the Methods section below [70], is used. The model takes into account only the contributions of the residues next to the cleavage site: C-terminus and the next aa. Two thresholds, 0.0 and 0.1, can be used here. Threshold 0.0 is recommended for alleles which prefer Phe or Trp at the C-terminus: HLA-A*24, HLA-B*07, HLA-B*27, HLA-B*35, HLA-B*51 and HLA-B*53. The epitopes for other alleles are predicted accurately at a threshold of 0.1. This initial step has a powerful filtering ability: between one half and two thirds of the true negatives were eliminated by this step. The "cleaved" peptides, present as nonamers, are then passed to the next filter: the TAP binding QM.

### EpiJen step two: TAP transport

The TAP binding QM also has been derived and tested previously [69]. A threshold of 5.00 is recommended for both fully and partially TAP-dependent alleles. Pro and Asp at anchor position 2 has a strong negative effect on TAP binding [69]. For that reason, a threshold of 3.0 is recommended for epitopes binding to HLA-B*07, HLA-B*35, HLA-B*40, HLA-B*44, HLA-B*51 and HLA-B*53. The filtering ability of the TAP step is low. Up to 10% of the true negatives are eliminated here. The "transported" peptides move to the next filter: MHC binding.

### EpiJen step three: MHC binding

EpiJen includes 18 QMs which can be used to predict binding to different HLA-A and B alleles. Certain QMs were developed for single alleles and others developed for allele families. QMs developed for whole supertypes were poorly predictive, especially for HLA-B supertypes. Some MHC models were derived previously [42,52,72], while others were developed for this study. The statistics of newly derived models are given in Tables 1 and 2. Quantitative data (continuous values like IC_50_s) were available for certain alleles, for the rest only sequences of binders were known (discontinuous values). As is described in the Methods section below, binding models based on continuous values were derived by multiple linear regression (MLR) (Table 1) and those based on discontinuous values by discriminant analysis (DA) (Table 2). "Leave-one-out" cross-validation tests indicate a higher predictive rate for the DA models (*AUC*_*ROC *_> 0.9; *accuracy *> 80%) than MLR models (*q*^2 ^≈ 0.5). The filtering ability of this step is significant: approximately 25–30% of the true negatives are eliminated here. The thresholds for this step are 0.5 for the DA models and 5.3 for MLR models. These thresholds can not be altered by the user. They seek to reduce the number of false positives in long protein sequences.

### EpiJen step four: epitope selection

All peptides which are presented by MHCs on the cell surface after being cleaved by the proteasome and transported by TAP could potentially be T cell epitopes. However, only a small number of all possible epitopes are actually immunogenic. To reduce the number of false positives we tested different thresholds, which we defined as percentages of available peptides sourced by one protein. The top 5% threshold performed best, giving 85% sensitivity; we recommend it and use it as a default value for this step. Optional are thresholds 2, 3 and 4%.

### External validation

A set of 160 epitopes and their source proteins were collected from AntiJen [73]. They were restricted by the human MHC allele families: HLA-A*01, HLA-A*02, HLA-A*03, HLA-A*11, HLA-A*24, HLA-A*33, HLA-A*68, HLA-B*07, HLA-B*27, HLA-B*35, HLA-B*40, HLA-B*44, HLA-B*51 and HLA-B*53. Six epitopes were promiscuous. Only proteins consisting of less than 1000 amino acids were used in the study. The thresholds were selected as follows: at step 1 (proteasome cleavage) a value of 0.0 was chosen for HLA-A*24, HLA-B*07, HLA-B*27, HLA-B*35, HLA-B*51 and HLA-B*53, and 0.1 for the rest; at step 2 (TAP transport) a value of 3.0 for HLA-B*07, HLA-B*35, HLA-B*51 and HLA-B*53, and 5.0 for the rest; at step 3 (MHC binding) a value of 0.5 was selected for HLA-A*24, HLA-B*27, HLA-B*40 and HLA-B*44, and 5.3 for the rest. For the final step (epitope selection) four thresholds were tested: top 2% to 5%. As the number of non-epitopes generated from each protein was significantly higher than the number of epitopes, only two parameters – *sensitivity *((true positives/(true positives + false negatives)) and *positive predictive value *(*PPV*) ((true positives/(true positives + false positives)) – were used for comparison. Parameters *accuracy *((true positives + true negatives)/total) and *specificity *((true negatives/(true negatives + false positives)) could be misleading. If 98% of the peptides in one source protein are non-epitopes, a model that simply predicts everything as non-epitope will not be very useful, yet it will nonetheless have an overall *accuracy *of 98% and a *specificity *of 100%.

**Figure 2:**
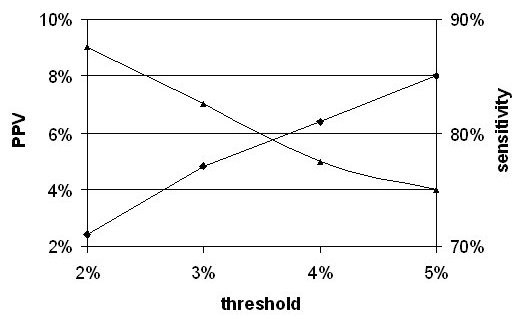
External validation of EpiJen. *Sensitivity *(-◆-) and *PPV *(-▲-) are given at different thresholds.

The true positives were 141 (5% cutoff), 132 (4% cutoff), 123 (3% cutoff) and 114 (2% cutoff). False negatives were 25, 34, 43 and 52, while the false positives decreased from 2743 to 2173, 1618 and 1060, respectively. The parameter *sensitivity *varies from 69% (at 2% cutoff) to 85% (at 5% cutoff) (Figure 2). The parameter *PPV *diminishes from 10% (at 2% cutoff) to 5% (at 5% cutoff). Thus, our tests indicate that a 5% threshold at the final epitope selection step is sufficient to generate an 85% epitope prediction. This means that by using EpiJen, one need only test 5% of the whole sequence in order to predict 85% of available epitopes. Detailed results from the external validation are given in Additional File 1, which is provided as Additional Material.

### Comparison with SMM, NetCTL and WAPP

The ability of EpiJen to predict T cell epitopes was tested on a set of known T cell epitopes, which belonged to 12 HIV proteins, and the resulting predictions were compared with those made by SMM, NetCTL and WAPP. The comparisons were made in conditions close to those used by experimental immunologist: the complete sequence of a protein of interest is submitted to an available web server and the results recorded. NetCTL and WAPP predictions were made using default thresholds. The top 5% best predicted peptides were selected as a threshold for SMM and EpiJen. As WAPP only predicts peptides binding only to HLA-A*01, HLA-A*02, HLA-A*03 and HLA-B*27, but SMM does not predict HLA-B*27 binding, the epitopes used in the test set were restricted to the set of alleles common to all four programs: HLA-A*01, HLA-A*02 and HLA-A*03. As described in Methods, a set of 99 epitopes was compiled: 4 peptides binding to HLA-A*01, 66 to HLA-A*02 and 29 to HLA-A*03. Three peptides were promiscuous, binding to both HLA-A*02 and HLA-A*03. The predictions were compared in terms of *sensitivity *and *PPV*.

EpiJen recognized 61 out of 99 epitopes (62%*sensitivity*), followed by SMM with 57 (58%*sensitivity*), NetCTL with 49 (50%*sensitivity*), and WAPP with 33 (33%*sensitivity*) (Table 3). The *PPV*s were low for all of the four methods: 21% for NetCTL, 17% for both EpiJen and WAPP, and 16% for SMM. Detailed results from this comparative study are given in Additional File 2, which is provided as Additional Material.

## Discussion

EpiJen is a server for multistep T cell epitope prediction. The principal steps in the antigen processing pathway are modeled by a set of different QMs. The models are applied successively, eliminating a proportion of negatives at each stage. Proteasome cleavage (step 1) and TAP transport (step 2) models are applied to all alleles. MHC binding (step 3) is more specific. Several models are included here. Some, like HLA-A*0101, HLA-A*0201, HLA-A*0202, HLA-A*0203, HLA-A*0206, HLA-A*0301, HLA-A*1101, HLA-A*3101, HLA-A*6801, HLA-A*6901 and HLA-A*3501, relate to single alleles. Others, such as HLA-A*24, HLA-B*07, HLA-B*27, HLA-B*40, HLA-B*44, HLA-B*51 and HLA-B*53, are valid for whole allele families. The final epitope selection step (step 4) reduces the number of false positives by selecting the top 5 to 2% of the best binders.

EpiJen combines several widely used methods in drug design [74,75], which have generally proven reliable and predictive. Moreover, the external validation of EpiJen and the comparison with other integrated methods showed that EpiJen performed best in terms of *sensitivity*. The number of peptides that have to be tested in order to reach 85% reliability is 5% of a protein sequence. According to Larsen et al. [65], NetCTL, SYFPEITHI and BIMAS achieve 85% reliability within the top 7%, 10% and 9%, respectively. The moderate performance of all integrated methods when used to predict the HIV epitope set may be due to the fact that all peptides available in the Los Alamos HIV Database were included, regardless of whether a peptide has been naturally processed or not. EpiJen excluded most of the false negative HIV epitopes after the proteasome cleavage step and only a few of them were predicted as nonbinders.

It is well known that "all models are wrong, yet some of them might be useful". The modeling process follows the accumulation of knowledge about a particular mechanism. As knowledge improves, so models improve. Antigen processing is a very complicated cascade of cellular events. It is clear that, for example, cleavage by the proteasome is only one event in antigen presentation: there are many more, and many of these are proteolytic. Analyses of peptide generation and T-cell epitopes expression in proteasome-inhibited cells suggest that cytoplasmic proteases other than proteasomes may also be involved in antigen processing pathway [76-78]. Tripeptidylpeptidase II (TPPII) was suggested to supply peptides because of its ability to cleave peptides in vitro and its upregulation in cells surviving partial proteasome inhibition [79]. Leucine aminopeptidase was found to generate antigenic peptides from N-terminally extended precursors [80]. Puromycin sensitive animopeptidase and bleomycin hydrolase were shown to trim N termini of synthetic peptides [81]. An enzyme located in the lumen in ER and called ERAAP (ER aminopeptidase associated with antigen processing) [82] or ERAP1 (ER aminopeptidase 1) [83,84], has been shown to be responsible for the final trimming of the N termini of peptides presented by MHC class I molecules. Recently, it was shown that within the proteasome, peptides could be formed from noncontiguous parts of the source protein [85,86]. The mechanism of this splicing is not fully understood. Currently there is insufficient quantitative data about the role of the above mentioned events to allow a precise bioinformatic evaluation of their impact on the antigen processing pathway. Overall, it is clear that, ultimately, many more pathways, involving many more stages, will need to incorporated into predictive methods in order to model it accurately; given current data, however, EpiJen represents the most accurate and parsimonious approach to antigen prediction.

Compared to other methods, EpiJen offers many potential advantages. First, a large quantity of experimental data (more than 2500 peptides) has been used to develop the models. Second, the additive method combines two well known, widely used methods in drug design [42], which have generally proven to be both reliable and predictive: the Free-Wilson method [74] and partial least squares (PLS) [75]. Finally, and most importantly, EpiJen uses its models as successive filters: negatives are eliminated at each step rather than their score being summed in order to exceed a global threshold. This is in contrast to alternative methods [64,65]. The combined score, as used by SMM and NetCTL, obscures the final result, because a low (or even negative) TAP and/or proteasome score could be compensated for by a high MHC score. The cellular antigen processing pathway, as modeled in EpiJen, works in a hierarchical or successive manner not in parallel. Peptides that have been eliminated at any of the steps do not continue to the next step. EpiJen is thus based on a more mechanistically meaningful model of antigen presentation than other available methods. EpiJen is both a more adaptable and a more flexible approach, which should prove a significant advantage as combination methods, such as this, evolve.

## Conclusion

EpiJen belongs to a new generation of integrated methods for T cell epitope prediction. It is an open system: new models will be included in the future, while old ones will be improved. Integrated methods aim to rationalize the process of epitope searching and accelerate epitope-based vaccine design. They possess significant potential for improving the predictive ability of *in silico *epitope identification by adding more features and new high quality experimental data.

## Methods

### Peptide datasets

More than 2500 peptides (nonamers) were used to develop models involved in EpiJen. Models for proteasome cleavage [70], TAP binding [69], HLA-A2 [71], HLA-A3 [52] and HLA-B35 [87] binding were derived previously. The majority of peptides were extracted from AntiJen [73], although the SYFPEITHI database [38] was also used. As has been described elsewhere [70], the training set for the proteasome cleavage model consisted of 489 naturally processed T-cell epitopes associated with HLA-A and HLA-B molecules and a test set of 231 peptides were used for external validation. All T-cell epitopes common to the two sets were first excluded from the training set. The training set for the TAP binding model included 163 poly-Alanine nonameric peptides and two test sets containing 85 peptides were used for external validation [69]. 1371 peptides were used for the development of the MHC binding models; the models were tested by "leave-one-out" cross-validation (LOO-CV).

### Additive method

The additive method [42] can be used to develop a QM for any particular peptide-protein interaction. Nonameric peptides are presented as a binary string of length 180 (9 positions × 20 amino acids). A term is equal to 1 when a particular amino acid at a particular position is present and 0 when it is absent. The dependent variable could take either continuous values, like logIC_50_, or discontinuous ones, such as binder or non-binder. When the dependent variable is continuous, multiple linear regression is used to derive the QM. In the case of a discontinuous dependent variable, models are derived by discriminant analysis. In both cases, the matrix is solved using partial least squares (PLS).

### Partial least squares (PLS)

PLS is a so called projection method [75], which can handle matrices with more variables than observations and with noisy and highly collinear data. In such cases, conventional statistical methods, such as multiple linear regression, produce over-fitted models, i.e. models that fit the training data well but are unreliable in prediction. PLS forms new variables, or principal components (PC), as linear combinations of the initial variables and then uses them to predict the dependent variable. The PLS method used in this study was implemented in SYBYL 6.9 [88]. The type of the dependent variable (continuous or discontinuous) determined the statistical method: linear regression or discriminant analysis.

### Multiple linear regression by partial least squares (MLR-PLS)

MLR-PLS requires at least 30 peptides with experimentally measured affinities (IC_50_) to generate good models. In this study MLR was applied only to models for binding to HLA-B7, HLA-B51 and HLA-B53 alleles. IC_50 _values were collected from AntiJen [73]. The optimal number of PC used to derive the model was defined as the number which lead to the highest cross-validated *q*^2 ^and/or the lowest standard error of prediction (*SEP*). The *q*^2 ^values were derived after "leave-one-out" cross-validation (LOO-CV). The non-cross-validated models were assessed by the explained variance *r*^2^, standard error of estimate (*SEE*), and *F*-ratio. The non-cross-validated models were used at EpiJen step 3.

### Discriminant analysis by partial least squares (DA-PLS)

Not enough quantitative data was found for peptide binding to alleles HLA-A24, HLA-B27, HLA-B40 and HLA-B44. Instead, sets of MHC binders for each allele were collected from AntiJen and SYFPEITHI. Each source protein was passed through the first two steps of EpiJen (proteasome cleavage and TAP binding) and only properly "cleaved" and "TAP-transported" peptides were selected. Among them the binders form a small set; the rest were considered as non-binders. The number of non-binders was significantly higher than that of binders (Table 2). In order to develop a reasonable model by DA, equivalent numbers of binders and non-binders are required. Otherwise, a model will represent an over- or under- estimation of amino acids contributions. The number of non-binders was reduced by the method of hierarchical clustering, as described below. This resulted in almost equivalent numbers of binders and non-binders for each allele family. The QMs were derived by PLS. The optimum number of PCs used do derive the model was defined as the number leading to the lowest standard error of prediction (*SEP*) after "leave-one-out" cross-validation (LOO-CV). The prediction rate of the models was measured by LOO-CV using Receiver Operating Characteristic (*ROC*) curves [89]. Two variables *sensitivity *(true positives/total positives) and *1-specificity *(false positives/total negatives) were calculated at different thresholds. The area under the curve (*AUC*_*ROC*_) is a quantitative measure of predictive ability and varies from 0.5 for a random prediction to 1.0 for a perfect prediction. Prediction a *ccuracy *((true positives + true negatives)/total) at a threshold 0.5, was also calculated. The non-cross-validated models were used at EpiJen step 3.

### Hierarchical clustering (HC)

Clustering is the process of dividing a set of entities into several subsets. Members of each subset are similar to each other but different from members of other subsets [90]. There have been numerous cluster methods described [91]. In the present study, HC, using the agglomerative algorithm [90], was applied. According to this algorithm, clusters are built from the bottom up, first by merging individual items into clusters, and then by merging clusters into superclusters, until the final merge brings all items into a single cluster. This method was applied as implemented in Sybyl 6.9 [88]. The distance between the clusters was calculated using the complete-linkage method, i.e. the distance between the most distant pair of data points in both clusters is taken into account.

In this study, HC reduced the number of non-binders for the DA-PLS models, i.e. models for peptide binding to HLA-A24, HLA-B27, HLA-B40 and HLA-B44. The last second or third level of the dendrogram, which contained a number of clusters close to the number of binders, was used. One peptide from each cluster was chosen at random to act as the exemplar for that cluster.

### Test sets

For the external validation of EpiJen, a set of 160 epitopes, and their source proteins were collected from AntiJen. The epitopes were not been used to develop any of the models included in EpiJen. Six epitopes were restricted to HLA-A*01, 58 to HLA-A*02, 4 to HLA-A*03, 6 to HLA-A*11, 25 to HLA-A*24, 1 to HLA-A*33, 1 to HLA-A*68, 12 to HLA-B*07, 10 to HLA-B*27, 25 to HLA-B*35, 1 to HLA-B*40, 7 to HLA-B*44, 6 to HLA-B*51 and 4 to HLA-B*53. To reduce the number of non-epitopes, only proteins consisting of less than 1000 amino acids were considered in the study. As the number of non-epitopes generated from one protein was significantly higher than the number of epitopes, only two parameters – *sensitivity *and *positive predictive value *((true positives/(true positives + false positives)) – were used for the assessment of program performance.

For the comparison between the integrated methods, a set of known HIV epitopes was collected from the CTL/CD8+ T cell epitope maps published in the HIV Molecular Immunology Database (last updates June 8, 2005) of the Los Alamos National Laboratory [92]. Epitopes are mapped to the HXB2 consensus protein sequence. Only nonamer epitopes, restricted by HLA-A*01, HLA-A*02 and HLA-A*03 at the serotype level, were included in the test set. Epitopes which spanned a protein boundary were not considered. The final set consisted of 99 epitopes from 12 source proteins. Proteins p2p7p1p6 and gag/pol TF did not have epitopes of interest and were not used. Four epitopes were restricted by HLA-A*01, 66 – by HLA-A*02 and 29 – by HLA-A*03. Three of the epitopes were promiscuous. Parameters *sensitivity *and *positive predictive value *were used to assess the results.

### EpiJen Server

The EpiJen server is implemented in Perl, with an interface written in HTML. EpiJen identifies epitopes from nucleic acid and protein sequences, using the four-stage model described above, with options to vary the relevant requisite parameters, such as selected MHC allele and individual model thresholds. Prediction from nucleic acid allows for 3 or 6 frame translation, as recently it has become known that many antigenic peptides emerge from cryptic translational reading frames [93,94], as well as through post-translational splicing [85,86], amongst other non-canonical expression mechanisms.

## Authors' contributions

IAD derived and tested the models included in this study. PG designed and implemented the web server. DRF supervised the project and contributed relevant bioinformatics knowledge. All authors have read and approved the final manuscript.

## Supplementary Material

Additional File 1 A .xls file containing the results from the external validation. A set of 160 epitopes, belonging to HLA-A*01, HLA-A*02, HLA-A*03, HLA-A*11, HLA-A*24, HLA-A*33, HLA-A*68, HLA-B*07, HLA-B*27, HLA-B*35, HLA-B*40, HLA-B*44, HLA-B*51 and HLA-B*53 allele families, and their source proteins (swiss-prot code) are given. Values for true positives (TP), false negatives (FN), false positives (FP), sensitivity and positive predictive value (PPV) are shown at different thresholds: 5%, 4%, 3% and 2%. Click here for file

Additional File 2 A .xls file containing the results from the validation on HIV test set. Twelve HIV proteins carrying 99 T cell epitopes belonging to HLA-A*01, HLA-A*02 and HLA-A*03 allele families were submitted to four different web servers for multistage T cell epitope prediction – NetCTL, EpiJen, WAPP and SMM – and detailed results for each protein are given. Click here for file

## Figures and Tables

**Table 1:** Statistics of the MHC models (EpiJen step 3), derived in this study by MLR-PLS.

Model	n	*q*^2^	*PC*	*SEP*	*r*^2^	*SEE*	*F*-ratio
HLA-B*07 binding	77	0.536	6	0.750	0.980	0.156	567.597
HLA-B*51 binding	69	0.495	4	0.775	0.959	0.221	371.878
HLA-B*53 binding	55	0.439	5	0.898	0.972	0.200	342.130

**Table 2:** Statistics of the MHC models (EpiJen step 3), derived in this study by DA-PLS.

Model	binders	non-binders	non-binders after HC selection	"leave-one-out" cross-validation	
				
				*AUC*_*ROC*_	*accuracy *at threshold 0.5
HLA-A*24 binding	72	2109	67	0.949	0.885
HLA-B*27 binding	68	877	61	0.988	0.899
HLA-B*40 & B*44 binding	20+19	619+555	19+18	0.916	0.842

**Table 3:** Comparison between EpiJen, NetCTL, WAPP and SMM on HIV epitopes, binding to HLA-A*01, HLA-A*02 and HLA-A*03.

	p17	p24	protease	RT	integrase	rev	tat	vif	vpr	vpu	gp160	nef	total
A*01	1	0	0	0	0	1	0	0	0	0	1	1	4
A*02	2	9	2	11	3	3	0	3	4	1	20	8	66
A*03	2	2	1	9	2	2	1	2	0	0	4	4	29
total	5	11	3	20	5	6	1	5	4	1	25	13	99
EpiJen													
TP	5	7	2	14	2	5	1	3	2	0	14	6	61
FN	0	4	1	6	3	1	0	2	2	1	11	7	38
FP	16	17	8	42	26	13	4	17	3	4	115	24	289
*Sensitivity *%	100	64	67	70	40	83	100	60	50	0	56	46	**62**
*PPV *%	24	29	20	25	7	28	20	15	40	0	11	20	**17**
NetCTL													
TP	4	6	1	10	3	3	1	4	1	0	12	4	49
FN	1	5	2	10	2	3	0	1	3	1	13	9	50
FP	8	10	6	39	22	5	7	11	4	2	60	14	188
*Sensitivity *%	80	55	33	50	60	50	100	80	25	0	48	31	**50**
*PPV *%	33	38	14	20	12	38	13	27	20	0	17	22	**21**
WAPP													
TP	2	2	2	7	1	4	0	3	3	0	8	1	33
FN	3	9	1	13	4	2	1	2	1	1	17	12	66
FP	8	6	4	40	13	6	2	13	0	6	61	7	166
*Sensitivity *%	40	18	67	35	20	67	0	60	75	0	32	8	**33**
*PPV *%	20	25	33	15	7	40	0	19	100	0	12	13	**17**
SMM													
TP	3	6	2	15	5	4	0	3	2	0	14	3	57
FN	2	5	1	5	0	2	1	2	2	1	11	10	42
FP	15	18	8	41	23	14	5	17	3	4	115	27	290
*Sensitivity *%	60	55	67	75	100	67	0	60	50	0	56	23	**58**
*PPV *%	17	25	20	27	18	22	0	15	40	0	11	10	**16**

## References

[B1] Sette A, Newman M, Livingston B, McKinney D, Sidney J, Ishioka G, Tangri S, Alexander J, Fikes J, Chestnut R (2002). Optimizing vaccine design for cellular processing, MHC binding and TCR recognition. Tissue Antigens.

[B2] Craiu A, Akopian T, Goldberg A, Rock KL (1997). Two distinct proteolytic processes in the generation of a major histocompatibility complex class I-presented peptide. Proc Natl Acad Sci USA.

[B3] Mo XY, Cascio P, Lemerise K, Goldberg AL, Rock K (1999). Distinct proteolytic processes generate the C and N termini of MHC class I-binding peptides. J Immunol.

[B4] Serwold T, Shastri N (1999). Specific proteolytic cleavages limit the diversity of the pool of peptides available to MHC class I molecules in living cells. J Immunol.

[B5] Cascio P, Hilton C, Kisselev AF, Rock KL, Goldberg AL (2001). 26S proteasomes and immunoproteasomes produce mainly N-extended versions of an antigenic peptide. EMBO J.

[B6] Orlowski M, Michaud C (1989). Pituitary multicatalytic proteinase complex. Specificity of components and aspects of proteolitic activity. Biochemistry.

[B7] Djaballah H, Harness JA, Savory PJ, Rivett AJ (1992). Use of serine-protease inhibitors as probes for the different proteolytic activities of the rat liver multicatalitic proteinase complex. Eur J Biochem.

[B8] Orlowski M, Cardozo C, Michaud C (1993). Evidence for the presence of five distinct proteolytic components in the pituitary multicatalytic proteinase complex. Properties of two components cleaving bonds on the carboxy side of branched chain and small neutral amino acids. Biochemistry.

[B9] Tanaka K, Kasahara M (1998). The MHC class I ligand-generating system: roles of immunoproteasomes and the interferon-γ-inducible proteasome activator PA28. Immunol Rev.

[B10] Van den Eynde BJ, Morel S (2001). Differential processing of class-I-restricted epitopes by the standard proteasome and the immunoproteasome. Curr Opin Immunol.

[B11] Toes RE, Nussbaum AK, Degermann S, Schirle M, Emmerich NP, Kraft M, Laplace C, Zwinderman A, Dick TP, Muller J, Schonfisch B, Schmid C, Fehling HJ, Stevanovic S, Rammensee H-G, Schild H (2001). Discrete cleavage motifs of constitutive and immunoproteasomes revealed by quantitative analysis of cleavage products. J Exp Med.

[B12] Rammensee H-G, Friede T, Stevanović S (1995). MHC ligands and peptide motifs: first listing. Immunogenetics.

[B13] Monaco J, Cho S, Attaya M (1990). Transport protein genes in the murine MHC – possible implications for antigen processing. Science.

[B14] Meyer TH, van Endert PM, Uebel S, Ehring B, Tampé R (1994). Functional expression and purification of the ABC transporter complex associated with antigen processing (TAP) in insect cells. FEBS Lett.

[B15] Müller KM, Ebensperger C, Tampé R (1994). Nucleotide binding to the hydrophilic C-terminal domain of the transporter associated with antigen processing (TAP). J Biol Chem.

[B16] Schumacher TNM, Kantesaria DV, Heemels MT, Ashton-Rickardt PG, Shepherd JC, Früh K, Yang Y, Peterson PA, Tonegawa S, Ploegh HL (1994). Peptide length and sequence specificity of the mouse TAP1/TAP2 translocator. J Exp Med.

[B17] Lautscham G, Rickinson A, Blake N (2003). TAP-independent antigen presentation on MHC class I molecules: lessons from Epstein-Barr virus. Microbes Infect.

[B18] Brusic V, van Endert P, Zeleznikow J, Daniel S, Hammer J, Petrovsky N (1998). A neural network model approach to the study of human TAP transporter. In Silico Biology.

[B19] de la Salle H, Houssaint E, Peyrat MA, Arnold D, Salamero J, Pinczon D, Stevanovic S, Bausinger H, Fricker D, Gomard E, Biddison W, Lehner P, UytdeHaag F, Sasporte M, Donato L, Rammensee HG, Cazenave JP, Hanau D, Tongio MM, Bonneville M (1997). Human peptide transporter deficiency: importance of HLA-B in the presentation of TAP-independent EBV antigens. J Immunol.

[B20] Mormung F, Neefjes JJ, Hämmerling GJ (1994). Peptide selection by MHC-encoded TAP transporters. Curr Opin Immunol.

[B21] Henderson RA, Michel H, Sakaguchi K, Shabanowitz J, Appella E, Hunt DF, Engelhard VH (1992). HLA-A2.1-associated peptides from a mutant cell line: a second pathway of antigen presentation. Science.

[B22] Guéguen M, Biddison W, Long EO (1994). T cell recognition of an HLA-A2-restricted epitope derived from a cleaved signal sequence. J Exp Med.

[B23] Smith KD, Lutz CT (1996). Peptide-dependent expression of HLA-B7 on antigen processing-deficient T2 cells. J Immunol.

[B24] Khanna R, Burrows SR, Moss DJ, Silins SL (1996). Peptide transporter (TAP-1 and TAP-2)-independent endogenous processing of Epstein-Barr virus (EBV) latent membrane protein 2A: implications for cytotoxic T-lymphocyte control of EBV-associated malignancies. J Virol.

[B25] Janeway CA, Travers P, Walport M, Capra JD (1999). Immunobiology The immune system in health and desease.

[B26] Saper MA, Bjorkman PJ, Wiley DC (1991). Refined structure of the human histocompatibility antigen HLA-A2 at 2.6 A resolution. J Mol Biol.

[B27] Smith KJ, Reid SW, Harlos K, McMichael AJ, Stuard DI, Bell JI, Jones EY (1996). Bound water structure and polymorphic amino acids act together to allow the binding of different peptides to MHC class I HLA-B53. Immunity.

[B28] Fan QR, Wiley DC (1999). Structure of human histocompatibility leukocyte antigen (HLA)-Cw4, a ligand for the KIR2D natural killer cell inhibitory receptor. J Exp Med.

[B29] Flower DR (2003). Towards *in silico *prediction of immunogenic epitopes. Trends Immunol.

[B30] Golgberg AL, Cascio P, Saric T, Rock KL (2002). The importance of the proteasome and subsequent proteolitic steps in the generation of antigenic peptides. Mol Immunol.

[B31] Schirle M, Weinschenk T, Stevanović S (2001). Combining computer algorithms with experimental approaches permits the rapid and accurate identification of T cell epitopes from defined antigens. J Immunol Methods.

[B32] DeLisi C, Berzofski JA (1985). T-cell antigenic sites tend to be amphipathic structures. Proc Natl Acad Sci USA.

[B33] Rothbard JB, Taylor WR (1988). A sequence pattern common to T cell epitopes. EMBO J.

[B34] Meister GE, Roberts CG, Berzofsky JA, De Groot AS (1995). Two novel T cell epitope prediction algorithms based on MHC-binding motifs; comparison of predicted and published epitopes from Mycobacterium tuberculosis and HIV protein sequences. Vaccine.

[B35] Deavin AJ, Auton TR, Greaney PJ (1996). Statistical comparison of established T cell epitope predictors against a large database of human and murine antigens. Mol Immunol.

[B36] Flower DR, Doytchinova IA, Paine K, Taylor P, Blythe MJ, Lamponi D, Zygouri C, Guan P, McSparron H, Kirkbride H, Flower DR (2002). Computational Vaccine Design. Drug Design Cutting Edge Approaches.

[B37] Sette A, Buus E, Appella JA, Smith R, Chesnut R, Miles C, Colon SM, Grey HM (1989). Prediction of major histocompatibility complex binding regions of protein antigens by sequence pattern analysis. Proc Nat Acad sci USA.

[B38] Rammensee H, Bachmann J, Emmerich NP, Bachor OA, Stevanovic S (1999). SYFPEITHI: database for MHC ligands and peptide motifs. Immunogenetics.

[B39] Reche PA, Glutting JP, Reinherz EL (2002). Prediction of MHC class I binding peptides using profile motifs. Hum Immunol.

[B40] Reche PA, Glutting JP, Reinherz EL (2004). Enhancement to the RANKPEP resource for the prediction of peptide binding to MHC molecules using profiles. Immunogenetics.

[B41] Marshall KW, Wilson KJ, Liang J, Woods A, Zaller D, Rothbard JB (1995). Prediction of peptide affinity to HLA-DRB1*0401. J Immunol.

[B42] Doytchinova IA, Blythe MJ, Flower DR (2002). Additive method for the prediction of protein-peptide binding affinity. Application to the MHC class I molecule HLA-A*0201. J Proteome Res.

[B43] Doytchinova IA, Flower DR (2003). Towards the *in silico *identification of class II restricted T cell epitopes: a partial least squares iterative self-consistent algorithm for affinity prediction. Bioinformatics.

[B44] Bisset LR, Fierz W (1993). Using a neutral network to identify potential HLA-DR1 binding sites within proteins. J Mol Recognit.

[B45] Gulukota K, DeLisi C (2001). Neural network method for predicting peptides that bind major histocompatibility complex molecules. Methods Mol Biol.

[B46] Honeyman MC, Brusic V, Stone NL, Harrison LC (1998). Neural network-based prediction of candidate T-cell epitopes. Nat Biotechnol.

[B47] Brusic V, Rudy G, Honeyman G, Hammer J, Harrison L (1998). Prediction of MHC class II-binding peptides using an evolutionary algorithm and artificial neural network. Bioinformatics.

[B48] Rognan D, Lauemoller SL, Holm A, Buus S, Tschinke V (1999). Predicting binding affinities of protein ligands from three-dimensional models: application to peptide binding to class I major histocompatibility proteins. J Med Chem.

[B49] Altuvia Y, Schueler O, Margalit H (1995). Ranking potential binding peptides to MHC molecules by a computational threading approach. J Mol Biol.

[B50] Doytchinova I, Flower DR (2001). Towards the quantitative prediction of T-cell epitopes: CoMFA and CoMSIA studies of peptides with affinity to class I MHC molecule HLA-A*0201. J Med Chem.

[B51] Doytchinova IA, Flower DR (2002). A Comparative Molecular Similarity Index Analysis (CoMSIA) study identifies an HLA-A2 binding supermotif. J Comput-Aid Mol Des.

[B52] Guan P, Doytchinova IA, Flower DR (2003). HLA-A3-supermotif defined by quantitative structure-activity relationship analysis. Protein Eng.

[B53] Dönnes P, Elofsson A (2002). Prediction of MHC class I binding peptides using SVMHC. BMC Bioinformatics.

[B54] Zhao Y, Pinilla C, Valmori D, Martin R, Simon R (2003). Application of support vector machines for T-cell epitope predictions. Bioinformatics.

[B55] Holzhutter HG, Frommel C, Kloetzel PM (1999). A theoretical approach towards the identification of cleavage-determining amino acid motifs of the 20S proteasome. J Mol Biol.

[B56] Kuttler C, Nussbaum AK, Dick TP, Rammensee H-G, Schild H, Hadeler K-P (2000). An algorithm for the prediction of proteasome cleavages. J Mol Biol.

[B57] Keşmir C, Nussbaum AK, Schild H, Detours V, Brunak S (2002). Prediction of proteasome cleavage motifs by neural networks. Protein Eng.

[B58] Uebel S, Meyer TH, Kraas W, Kienle S, Jung G, Wiesmüller KH, Tampé R (1995). Requirements for peptide binding to the human transporter associated with antigen processing revealed by peptide scans and complex peptide libraries. J Biol Chem.

[B59] Daniel S, Brusic V, Caillat-Zucman S, Petrovsky N, Harrison L, Riganelli D, Sinigaglia F, Gallazzi F, Hammer J, van Endert PM (1998). Relationship between peptide selectivities of human transporters associated with antigen processing and HLA class I molecules. J Immunol.

[B60] Peters B, Bulik S, Tampé R, van Endert PM, Holzhütter HG (2003). Identifying MHC class I epitopes by predicting the TAP transport efficiency of epitope precursors. J Immunol.

[B61] Bhasin M, Raghava GPS (2004). Analysis and prediction of affinity of TAP binding peptides using cascade SVM. Protein Sci.

[B62] Hakenberg J, Nussbaum AK, Schild H, Rammensee HG, Kuttler C, Holzhutter HG, Kloetzel PM, Kaufmann SHE, Mollenkopf HJ (2003). MAPPP: MHC class I antigenic peptide processing prediction. Appl Bioinformatics.

[B63] Tenzer S, Peters B, Bulik S, Schoor O, Lemmel C, Schatz MM, Kloetzel PM, Rammensee HG, Schild H, Holzhütter HG (2005). Modeling the MHC class I pathway by combining predictions of proteasomal cleavage, TAP transport and MHC class I binding. Cell Mol Life Sci.

[B64] Peters B, Sette A (2005). Generating quantitative models describing the sequence specificity of biological process with the stabilized matrix method. BMC Bioinformatics.

[B65] Larsen MV, Lundegaard C, Lamberth K, Buus S, Brunak S, Lund O, Nielsen M (2005). An integrative approach to CTL epitope prediction: A combined algorithm integrating MHC class I binding, TAP transport efficiency, and proteasomal cleavage predictions. Eur J Immunol.

[B66] Dönnes P, Kohlbacher O (2005). Integrated modeling of the major events in the MHC class I antigen processing pathway. Protein Sci.

[B67] Guan P, Doytchinova I, Hattotuwagama C, Flower DR (2006). MHCPred 2.0, an updated quantitative T cell epitope prediction server. Appl Bioinformatics.

[B68] Doytchinova IA, Walshe VA, Jones NA, Gloster SE, Borrow P, Flower DR (2004). Coupling in silico and in vitro analysis of peptide-MHC binding: A Bioinformatic approach enabling prediction of superbinding peptides and anchorless epitopes. J Immunol.

[B69] Doytchinova IA, Hemsley S, Flower DR (2004). Transporter associated with antigen processing preselection of peptides binding to the MHC: A Bioinformatic evaluation. J Immunol.

[B70] Doytchinova IA, Flower DR (2006). Class I T cell epitope prediction: improvements using a combination of Proteasome cleavage, TAP affinity, and MHC binding. Mol Immun.

[B71] EpiJen server for T cell epitope prediction. http://www.jenner.ac.uk/EpiJen.

[B72] Doytchinova IA, Flower DR (2003). The HLA-A2-supermotif: A QSAR definition. Org & Biomol Chem.

[B73] Toseland CP, Taylor DJ, McSparron H, Hemsley SL, Blythe MJ, Paine K, Doytchinova IA, Guan P, Hattotuwagama CK, Flower DR (2005). AntiJen: a quantitative immunology database integrating functional, thermodynamic, kinetic, biophysical, and cellular data. Immunome Res.

[B74] Free SM, Wilson JW (1964). A mathematical contribution to structure – activity studies. J Med Chem.

[B75] Wold S, Van der Waterbeemd H, Weinheim VCH (1995). PLS for Multivariate Linear Modeling. Chemometric Methods in Molecular Design.

[B76] Vinitsky A, Anton LC, Snyder HL, Orlowski M, Bennink JR, Yewdell JW (1997). The generation of MHC class I-associated peptides is only partially inhibited by proteasome inhibitors: involvement of nonproteasomal cytosolic proteases in antigen processing. J Immunol.

[B77] Luckey CJ, King GM, Marto JA, Venketeswaran S, Maier BF, Crotzer VL, Colella TA, Shabanowitz J, Hunt DF, Engelhard VH (1998). Proteasomes can either generate or destroy MHC class I epitopes: evidence for nonproteosomal epitope generation in the cytosol. J Immunol.

[B78] Luckey CJ, Marto JA, Partridge M, Hall E, White FM, Lippolis JD, Shabanowitz J, Hunt DF, Engelhard VH (2001). Differences in the expression of human class I MHC alleles and their associated peptides in the presence of proteasome inhibitors. J Immunol.

[B79] Geier E, Pfeifer G, Wilm M, Lucchiari-Hartz M, Baumeister W, Eichmann K, Niedermann G (1999). A giant protease with potential to substitute for some functions of the proteasome. Science.

[B80] Beninga J, Rock KL, Goldberg AL (1998). Interferon-gamma can stimulate post-proteasomal trimming of the N terminus of an antigenic peptide by inducing leucine aminopeptidase. J Biol Chem.

[B81] Stoltze L, Schirle M, Schwarz G, Schroeter C, Thompson MW, Hersh LB, Kalbacher H, Stevanović S, Rammensee H-G, Schild H (2000). Two new proteases in the MHC class I processing pathway. Nat Immunol.

[B82] Serwold T, Gonzalez F, Kim J, Jacob R, Shastri N (2002). ERAAP customizes peptides for MHC class I molecules in the endoplasmic reticulum. Nature.

[B83] Saric T, Chang S-C, Hattori A, York IA, Markant S, Rock KL, Tsujimoto M, Goldberg AL (2002). An IFN-γ-induced aminopeptidase in the ER, ERAPI, trims precursors to MHC class I-presented peptides. Nat Immunol.

[B84] York IA, Chang S-C, Saric T, Keys JA, Favreau JM, Goldberg AL, Rock KL (2002). The ER aminopeptidase ERAPI enhances or limits antigen presentation by trimming epitopes to 8–9 residues. Nat Immunol.

[B85] Hanada K, Yewdell JW, Yang JC (2004). Immune recognition of a human renal cancer antigen through post-translational protein splicing. Nature.

[B86] Vigneron N, Stroobant V, Chapiro J, ooms A, Degiovanni G, Morel S, van der Bruggen P, Boon T, van den Eynde BJ (2004). An antigenic peptide produced by peptide splicing in the proteasome. Science.

[B87] Hattotuwagama CK, Guan P, Doytchinova IA, Zygouri C, Flower DR (2004). Quantitative online prediction of peptide binding to the major histocompatibility complex. J Mol Graph Model.

[B88] (2004). SYBYL 6.9.

[B89] Bradley AP (1997). The use of the area under the ROC curve in the evaluation of machine learning algorithms. Pattern Recognition.

[B90] Barnard JM, Downs GM (1992). Clustering of chemical structures on the basis of two-dimensional similarity measures. J Chem Inf Comput Sci.

[B91] Brown RD, Martin YC (1996). Use of structure-activity data to compare structure-based clustering methods and descriptors for use in compound selection. J Chem Inf Comput Sci.

[B92] HIV Molecular Immunology Database. http://www.hiv.lanl.gov.

[B93] Schirmbeck R, Riedl P, Fissolo N, Lemonnier FA, Bertoletti A, Reimann J (2005). Translation from cryptic reading frames of DNA vaccines generates an extended repertoire of immunogenic, MHC class I-restricted epitopes. J Immunol.

[B94] Schwab SR, Shugart JA, Horng T, Malarkannan S, Shastri N (2004). Unanticipated Antigens: Translation Initiation at CUG with Leucine. PLoS Biol.

